# Systemic delivery of engineered compact AsCas12f by a positive-strand RNA virus vector enables highly efficient targeted mutagenesis in plants

**DOI:** 10.3389/fpls.2024.1454554

**Published:** 2024-09-10

**Authors:** Kazuhiro Ishibashi, Satoru Sukegawa, Masaki Endo, Naho Hara, Osamu Nureki, Hiroaki Saika, Seiichi Toki

**Affiliations:** ^1^ Division of Plant Molecular Regulation Research, Institute of Agrobiological Sciences, National Agriculture and Food Research Organization, Tsukuba, Japan; ^2^ Division of Crop Genome Editing Research, Institute of Agrobiological Sciences, National Agriculture and Food Research Organization, Tsukuba, Japan; ^3^ Department of Biological Sciences, Graduate School of Science, The University of Tokyo, Bunkyo-ku, Japan; ^4^ Graduate School of Nanobioscience, Yokohama City University, Yokohama, Japan; ^5^ Kihara Institute for Biological Research, Yokohama City University, Yokohama, Japan; ^6^ Department of Life Science, Faculty of Agriculture, Ryukoku University, Otsu, Japan

**Keywords:** AsCas12f, *Nicotiana benthamiana*, potato virus X, *Oryza sativa*, targeted mutagenesis

## Abstract

Because virus vectors can spread systemically autonomously, they are powerful vehicles with which to deliver genome-editing tools into plant cells. Indeed, a vector based on a positive-strand RNA virus, potato virus X (PVX), harboring SpCas9 and its single guide RNA (sgRNA), achieved targeted mutagenesis in inoculated leaves of *Nicotiana benthamiana*. However, the large size of the *SpCas9* gene makes it unstable in the PVX vector, hampering the introduction of mutations in systemic leaves. Smaller Cas variants are promising tools for virus vector–mediated genome editing; however, they exhibit far lower nuclease activity than SpCas9. Recently, AsCas12f, one of the smallest known Cas proteins so far (one-third the size of SpCas9), was engineered to improve genome-editing activity dramatically. Here, we first confirmed that engineered AsCas12f variants including I123Y/D195K/D208R/V232A exhibited enhanced genome-editing frequencies in rice. Then, a PVX vector harboring this AsCas12f variant was inoculated into *N. benthamiana* leaves by agroinfiltration. Remarkably, and unlike with PVX-SpCas9, highly efficient genome editing was achieved, not only in PVX-AsCas12f-inoculated leaves but also in leaves above the inoculated leaf (fourth to sixth upper leaves). Moreover, genome-edited shoots regenerated from systemic leaves were obtained at a rate of >60%, enabling foreign DNA–free genome editing. Taken together, our results demonstrate that AsCas12f is small enough to be maintained in the PVX vector during systemic infection in *N. benthamiana* and that engineered AsCas12f offers advantages over SpCas9 for plant genome editing using virus vectors.

## 1 Introduction

Genome editing using sequence-specific nucleases (SSNs) enables the introduction of mutations into a target gene in plants. In many cases, conventional genetic transformation using *Agrobacterium* has been employed to introduce and express SSN-encoding genes. However, this method restricts the range of plant species to which genome editing can be applied as some species are recalcitrant to tissue culture and transformation (Altpeter et al., 2016). Direct introduction of SSNs such as the ribonucleoprotein complex of clustered regularly interspaced short palindromic repeats - CRISPR associated protein 9 (CRISPR-Cas9) into shoot apical meristems is one of the DNA/tissue culture-free genome-editing technologies available in plants (Imai et al., 2020). However, the preparation of seed embryos is laborious. The delivery of SSNs through virus vectors is an alternative method that does not require the long and labor-intensive process of tissue culture-mediated transformation, although the large size of SSN-encoding genes makes them prone to deletion from viral genomes, posing a significant obstacle to virus vector–mediated genome editing. Somatic and systemic genome editing is induced using negative-strand RNA virus vectors expressing Cas9 derived from *Streptococcus pyogenes* (SpCas9) (Ma et al., 2020; Liu et al., 2023). However, the genomic RNA of a negative-strand RNA virus is masked by the viral nucleocapsid protein throughout an infection cycle. As a result, it may not be suitable for embedding RNA elements, such as meristem-directed sequences, to introduce heritable mutations (Ellison et al., 2020). A potato virus X (PVX)–based vector (a positive-strand RNA virus) was able to express SpCas9 and induce targeted mutagenesis in inoculated leaves of *N. benthamiana* (Ariga et al., 2020) but cannot do so in systemic leaves due to instability of the large *SpCas9* gene.

Cas12f (formerly Cas14a) is a miniature Cas protein [400–700 amino acids; Harrington et al. (2018)] with a double-stranded DNA cleavage activity (Karvelis et al., 2020) that has been used for targeted mutagenesis in plants. Cas12f derived from an uncultured archaeon and *Syntrophomonas palmitatica* has been used for targeted mutagenesis in maize, rice, tobacco, and tomato (Bigelyte et al., 2021; Sukegawa et al., 2023; Gong et al., 2024; Tang et al., 2024). Cas12f protein derived from *Acidibacillus sulfuroxidans* (AsCas12f) comprises 422 amino acids, i.e., one-third the size of SpCas9 (1,368 amino acids) and recognizes 5′-TTR-3′ (R=A/G) as a protospacer-adjacent motif (PAM) (Bigelyte et al., 2021). This compact AsCas12f protein could be a candidate tool for virus vector–mediated targeted mutagenesis and could represent a long-awaited SSN for transformation-free genome editing in plants. However, no mutations were detected after application of AsCas12f to targeted mutagenesis using geminiviral DNA replicons in *N. benthamiana* (Gong et al., 2024). The AsCas12f protein and its sgRNA have been engineered to improve its genome-editing activity to levels comparable with those of SpCas9 (Hino et al., 2023; Wu et al., 2023a, b). Very recently, targeted mutagenesis using two engineered AsCas12f variants produced by Hino et al. (2023) was reported in rice (Ye et al., 2024).

In this study, we carefully compared targeted mutagenesis frequencies among six engineered AsCas12f variants produced by Hino et al. (2023) and Wu et al. (2023a) in rice and found that the D195K/D208R/V232A-type variants produced by Hino et al. (2023) were superior to others. Based on these results, we applied the engineered AsCas12f variant exhibiting enhanced genome-editing frequencies in rice to virus vector–mediated targeted mutagenesis in *N. benthamiana* and succeeded in improving targeted mutagenesis frequency significantly using a PVX vector.

## 2 Materials and methods

### 2.1 Oligonucleotides

The primers used in this study are listed in **Supplementary Table S1**.

### 2.2 Vector construction

**Figure 1:**
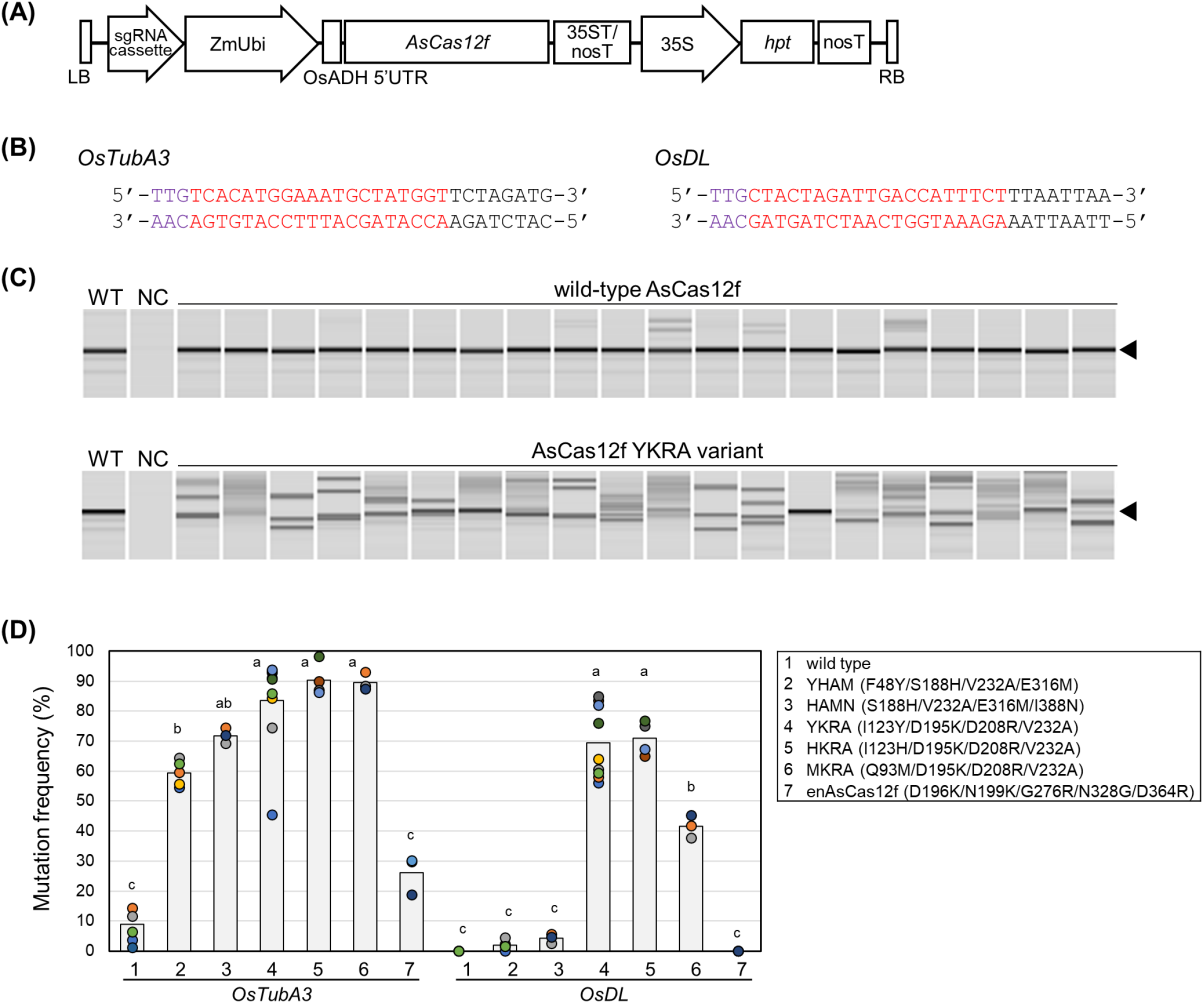
Genome editing using engineered AsCas12f in rice. **(A)** Schematic representation of binary vectors expressing AsCas12f and its sgRNA. **(B)** Target sequence of AsCas12f in *OsTubA3* and *OsDL*. Purple and red letters represent the PAM sequence and the target sequence, respectively. **(C)** Representative examples of heteroduplex mobility assay (HMA) to detect mutations in *OsTubA3* in calli transformed with wild-type AsCas12f and engineered AsCas12f YKRA variant. Triangles indicate the amplicon size in wild-type. WT, wild-type; NC, no template control. **(D)** Mutation frequency using engineered AsCas12f at the target site of *OsTubA3* and *OsDL* in transformed rice calli. #1, wild type AsCas12f; #2–6, AsCas12f variants produced by Hino et al. (2023); #7, enAsCas12f produced by Wu et al. (2023a). Circles and bars indicate the mutation frequency in each experiment and the average frequency, respectively. Different letters indicate significant differences (*P* < 0.05, Tukey’s test). Raw data are listed in **Supplementary Table S3**.

**Figure 2:**
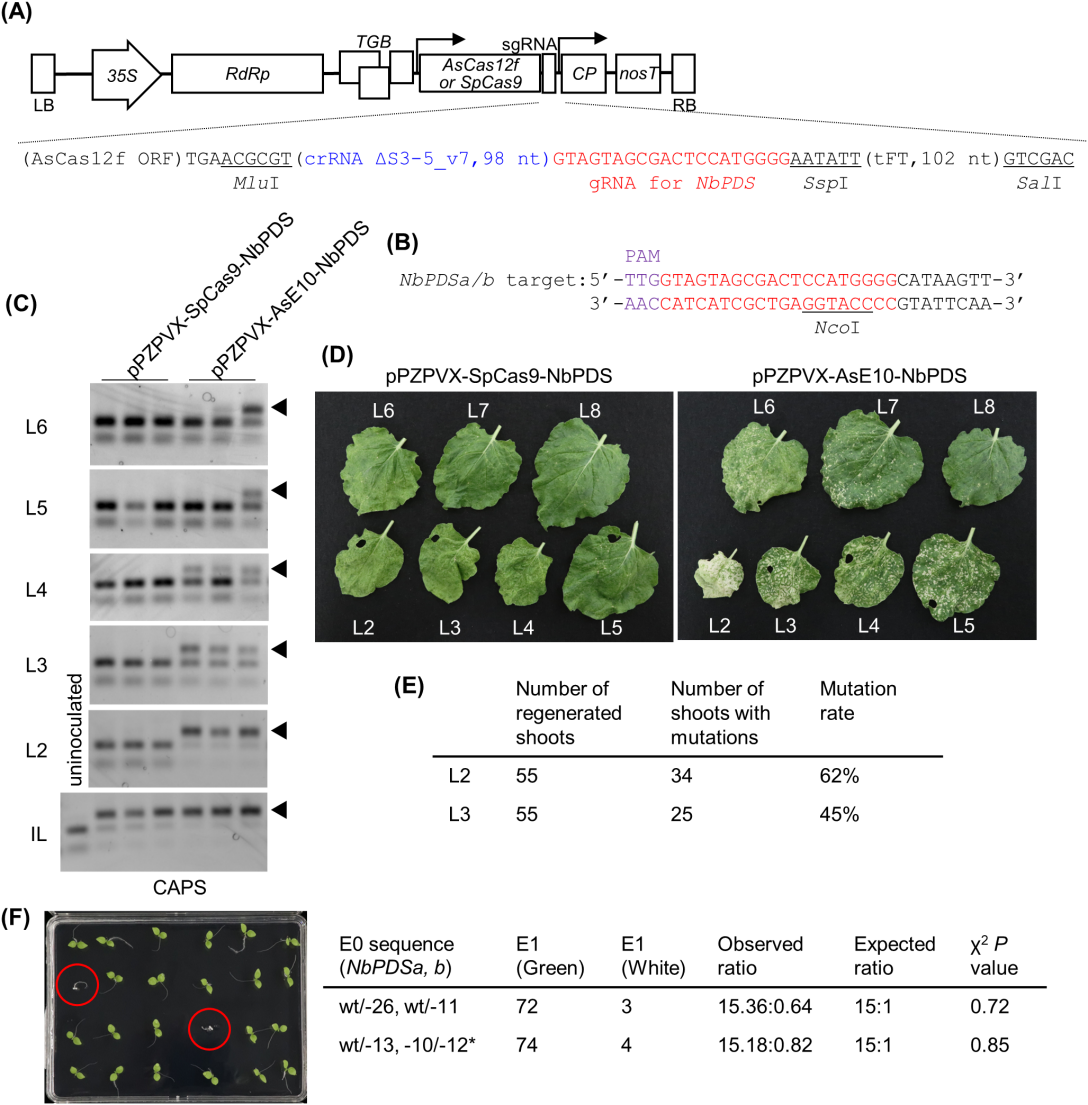
Genome editing using PVX vector expressing engineered AsCas12f in *N. benthamiana*. **(A)** Schematic representation of PVX vectors expressing AsCas12f or SpCas9. The nucleotide sequence around the sgRNA of pPZPVX-AsE10-NbPDS is shown below. The full nucleotide sequence is available in **Supplementary Table S2**. **(B)** Target sequence of AsCas12f in *NbPDS*. Purple and red letters represent the PAM sequence and the target sequence, respectively. **(C)** Detection of targeted mutations in PVX-AsCas12f or PVX-SpCas9-inoculated *N. benthamiana* plants by CAPS at 7 dpi for inoculated leaves (bottom panel) and 17 dpi for uninoculated upper leaves (upper panels). IL indicates inoculated leaves, and upper uninoculated leaves are shown as the leaf number counted from the inoculated leaf. Lanes represent independent plants. Similar results were obtained in five independent experiments, each involving inoculation of two or three plants. **(D)** Leaves of PVX-AsCas12f-NbPDS– or PVX-SpCas9-NbPDS–inoculated *N. benthamiana* plants at 27 dpi. **(E)** Summary of the mutation rates in shoots regenerated from the second (L2) or third (L3) upper leaves from PVX-AsCas12f-inoculated leaves. Mutations were detected by PCR and fragment analysis. **(F)** Inheritance of introduced mutations in the regenerated plants. Albino plants are marked by red circles. Asterisk indicates the 12-bp in-frame deletion that did not significantly impair protein function. The photograph shows 24 of the 72 plants from Line 1, with wt/-26 and wt/-11 mutations as indicated in the table on the right.

Rice codon– or Arabidopsis codon–optimized AsCas12f-coding sequence and sgRNA (Hino et al., 2023) including an OsU6 promoter were synthesized by GeneArt Gene Synthesis (Thermo Fisher Scientific). For rice genome editing, rice codon-optimized AsCas12f fused to a nuclear localization signal at the C terminus was replaced with SpCas9-NG in the vector pPZP ZmUbi-SpCas9-NG, yielding pPZP_ZmUbi_AsCas12f-Os C-NLS. To replace wild-type AsCas12f with six engineered AsCas12f variants, PCR-amplified fragments or synthesized DNA fragments of AsCas12f were inserted into *Pst*I/*Kpn*I or *Stu*I/*Sac*I-digested pPZP_ZmUbi_AsCas12f-Os C-NLS vector by an In-fusion reaction (TaKaRa). The annealed oligonucleotide pairs for the target sequences of *OsTubA3* and *OsDL* (**Supplementary Figure S1B**) were cloned into the *Bbs*I site in pUC19 AsCas12f-sgRNA vector by ligation reaction. The resultant sgRNA expression cassette for *OsTubA3* (Os03g0726100) or *OsDL* (Os03g0215200) was digested with *Asc*I/*Pac*I and inserted into the *Asc*I/*Pac*I site in the AsCas12f binary vectors by ligation (**Figures 1A, B**). For PVX-mediated *N. benthamiana* genome editing, the AsCas12f-coding sequence and the sgRNA were organized tandemly and cloned into pPZPVX301 (Ariga et al., 2020) to obtain pPZPVX-AsE10-NbPDS and pPZPVX-AsE10-NbTOM1 (**Figure 2A** and **Supplementary Figure S4**). Guide RNA sequences were designed to target all four alleles of the amphidiploid genome with appropriate restriction enzyme sites for cleaved amplified polymorphic sequences (CAPS). The nucleotide sequences of binary vectors are provided in **Supplementary Table S2**.

### 2.3 Genome editing of rice using *Agrobacterium*

Binary vectors were introduced into *Agrobacterium* strain EHA105 (Hood et al., 1993) by electroporation. The transformation procedure essentially followed that of previous studies (Toki, 1997; Sukegawa et al., 2023). One-month-old calli derived from mature seeds (cv. Nipponbare) were infected with *Agrobacterium* harboring a binary vector, and co-cultivated for 3 days at 23°C under constant dark. Infected calli were washed and selected on hygromycin-containing medium for 6 weeks, transferring to fresh medium every 2 weeks at 30°C under constant light. Transformed calli were cultured on regeneration medium and subsequently on hormone-free medium to obtain regenerated seedlings at 28°C under 16-h light/8-h dark conditions.

Genomic DNA was extracted from transformed calli (after 6 weeks of selection) and plants regenerated as described by Edwards et al. (1991). PCR was conducted with the KOD One PCR Master Mix (Toyobo), and primers are listed in **Supplementary Table S1** following the manufacturer’s protocols. Heteroduplex mobility assay (HMA) was performed by electrophoresis of the diluted PCR products on a microchip electrophoresis device, MultiNA (Shimadzu). Statistical analysis was performed using Tukey’s test using the program, MEPHAS (http://www.gen-info.osaka-u.ac.jp/MEPHAS/tukey.html). PCR products amplified from regenerated plants were used for Sanger sequence analysis. Sanger sequencing and fragment purification were performed using a BigDye Terminator v3.1 Cycle Sequencing Kit and a BigDye XTerminator Purification Kit (Thermo Fisher Scientific) following the manufacturer’s protocols. DNA sequences were determined using a 3500xL Genetic Analyzer (Thermo Fisher Scientific) and analyzed with SnapGene (GSL Biotech).

### 2.4 Genome editing of *N. benthamiana* using a PVX vector

Growth of *N. benthamiana*, inoculation of a PVX vector by agroinfiltration, and shoot regeneration from infected leaf tissues were performed as described previously (Ariga et al., 2020). DNA was extracted from agro-inoculated plants or regenerated shoots using the Kaneka Easy DNA Extraction Kit version 2 (Kaneka). PCR was performed with primers listed in **Supplementary Table S1**, and PCR products were digested with *Nco*I or *Kpn*I for CAPS of the target sites in *NbPDS* or *NbTOM1*, respectively, cloned for Sanger sequencing, or subjected to fragment analysis using SeqStudio and GeneMapper (Thermo Fisher Scientific). RNA was extracted from leaves using the RNeasy Plant Mini Kit (QIAGEN). PVX RNA was detected in total RNA by reverse transcription (RT)-PCR using the PrimeScript One-Step RT-PCR Kit (TaKaRa) for 40 reaction cycles.

## 3 Results and discussion

### 3.1 Comparison of genome-editing frequencies among AsCas12f variants in rice

We have previously established a reliable and efficient genome-editing system using SpCas9 (Mikami et al., 2015a, b). In addition, we succeeded in SpCas12f-mediated genome editing in rice (Sukegawa et al., 2023). This established rice genome-editing system was thought to be the best choice to evaluate genome-editing frequencies in AsCas12f variants. First, we compared targeted mutagenesis frequencies using six engineered AsCas12f variants [YHAM (F48Y/S188H/V232A/E316M), HAMN (S188H/V232A/E316M/I388N), YKRA (I123Y/D195K/D208R/V232A), HKRA (I123H/D195K/D208R/V232A), MKRA (Q93M/D195K/D208R/V232A) (Hino et al., 2023), and enAsCas12f (D196K/N199K/G276R/N328G/D364R) (Wu et al., 2023a)] in rice. Rice calli (cv. Nipponbare) were transformed via the *Agrobacterium*-mediated method with expression vectors harboring rice-codon-optimized AsCas12f driven by the maize ubiquitin promoter and its truncated sgRNA [ΔS3-5_v7; Hino et al. (2023)] including a 20-nt target sequence for *OsTubA3* and *OsDL* (**Figures 1A, B**). Six weeks after transformation, mutation frequencies were calculated as the ratio of mutated calli, in which heteroduplex bands were observed by HMA (**Figure 1C, D**). For both targets, mutation frequencies of the six engineered AsCas12f variants were higher than those obtained with wild-type AsCas12f (**Figure 1D**). Among six engineered AsCas12f variants, those produced by Hino et al. (2023) (**Figure 1D**, #2–6) showed higher mutation frequencies than the enAsCas12f produced by Wu et al. (2023a) (**Figure 1D**, #7). The mutation frequencies achieved in *OsTubA3* using engineered AsCas12f variants (YHAM, HAMN, YKRA, HKRA, and MKRA) were quite high (60%–90%) (**Figure 1D**). On the other hand, mutation frequencies achieved in *OsDL* using engineered AsCas12f variants (YKRA, HKRA, and MKRA) were ca. 70%, 70% and 40%, respectively, although the mutation frequency with engineered AsCas12f variants YHAM and HAMN was less than 5% (**Figure 1D**). These results suggest that D195K/D208R/V232A-type AsCas12f variants work better than S188H/V232A/E316M-type AsCas12f variants in targeted mutagenesis in rice as reported by Ye et al. (2024), although the genome-editing activities of S188H/V232A/E316M and D195K/D208R/V232A were comparable in human HEK293 cells (Hino et al., 2023). We obtained regenerated plants from calli transformed with AsCas12f variants harboring YHAM and YKRA mutations. As in our previous report of targeted mutagenesis via SpCas12f (Sukegawa et al., 2023), and a report of AsCas12f-mediated targeted mutagenesis (Ye et al., 2024), deletions ranging from several to dozens of base pairs were confirmed at the targeted site, some of which were thought to be derived from microhomology-mediated end-joining repair (**Supplementary Figure S1**). Unlike SpCas12f-mediated targeted mutagenesis (Sukegawa et al., 2023), biallelic regenerated plants in *OsTubA3* were obtained successfully using either engineered AsCas12f variant. Unfortunately, no mutated regenerated plants in *OsDL* were obtained using the YHAM variant. Overall, the engineered AsCas12f variant YKRA was considered a promising candidate for targeted mutagenesis using a virus vector.

### 3.2 Virus vector–mediated genome editing using AsCas12f in *N. benthamiana*

Next, we cloned the engineered, Arabidopsis codon–optimized, AsCas12f (YKRA variant) and the truncated sgRNA ΔS3-5_v7 (Hino et al., 2023) targeting the *N*. *benthamiana PDS* gene (*NbPDS*) into the PVX vector (**Figures 2A, B**). The resulting plasmid, pPZPVX-AsE10-NbPDS, was transformed into *Agrobacterium* and inoculated into the fifth true leaves of *N. benthamiana* plants by agroinfiltration. Highly efficient genome editing was detected in pPZPVX-AsE10-NbPDS–inoculated leaves, comparable with levels achieved using pPZPVX-Cas9-NbPDS encoding SpCas9 (Ariga et al., 2020), as monitored by CAPS (**Figure 2C**). No mutations were detected in uninoculated upper leaves of pPZPVX-Cas9-NbPDS–inoculated plants (**Figure 2C**). In contrast, targeted mutations were detected in the second to fourth upper leaves or higher, counting from the inoculated leaf, in pPZPVX-AsE10-NbPDS–inoculated plants (**Figure 2C**). Introduced mutations were confirmed by sequencing (**Supplementary Figure S2**). Photobleaching, suggestive of knockout of the *PDS* gene, was observed in systemic leaves of pPZPVX-AsE10-NbPDS–inoculated plants, whereas the proportion of white versus green areas decreased gradually in the higher leaves (**Figure 2D**). RNA was extracted from each leaf, and the presence of the inserted sequences in the PVX vectors was examined by RT-PCR. Amplified fragments with an expected length of the intact AsCas12f and sgRNA sequence (1.7 kbp) were detected in uninoculated (up to sixth) leaves of pPZPVX-AsE10-NbPDS–inoculated plants, whereas those of the SpCas9 and sgRNA sequence (4.4 kbp) were detected only in inoculated leaves, and smaller fragments (0.6–0.7 kbp) were detected in upper uninoculated leaves of pPZPVX-Cas9-NbPDS–inoculated plants (**Supplementary Figure S3**). Thus, AsCas12f is small enough to be maintained in the PVX vector during systemic infection in *N. benthamiana*. Another sgRNA targeting the *NbTOM1* gene introduced in the PVX vector also induced systemic mutations (**Supplementary Figure S4**). A PVX vector harboring a sgRNA for SpCas9 and a truncated *Flowering locus T* (*FT*) sequence induces heritable mutations in *N. benthamiana* (Uranga et al., 2021), and pPZPVX-AsE10-NbPDS had the truncated *FT* sequence by which we aimed to induce genome editing in germline cells (**Supplementary Table S2C**). However, no gene-edited progenies from seeds from pPZPVX-AsE10-NbPDS–inoculated plants were obtained under our conditions. For foreign DNA–free genome editing, shoots were regenerated from uninoculated upper leaves of pPZPVX-AsE10-NbPDS–inoculated plants. Approximately 60% of the shoots regenerated from the second upper leaves had mutations in *NbPDS* (**Figure 2E**)—an efficiency comparable with that of pPZPVX-Cas9-NbPDS–agroinoculated leaves (Ariga et al., 2020). Introduced mutations were inherited to the next generation according to Mendelian laws of inheritance (**Figure 2F**). Note that a previous study had revealed that PVX remains in most regenerated shoots but is not transmitted to the progeny (Ariga et al., 2020). Taken together, these results demonstrate that engineered AsCas12f offers advantages over SpCas9 for plant genome editing using virus vectors.

## 4 Conclusion

In conclusion, we have demonstrated the usefulness of engineered AsCas12f in targeted mutagenesis in rice and *Nicotiana* and succeeded in the significant improvement of targeted mutagenesis frequency using a PVX vector. The AsCas12f–virus vector system thus shows promise as a powerful tool in plant genome editing.

## Data Availability

The raw data supporting the conclusions of this article will be made available by the authors, without undue reservation.
