# Supplementary material for: Systemic delivery of engineered compact AsCas12f by a positive-strand RNA virus vector enables highly efficient targeted mutagenesis in plants
Source: Front Plant Sci. 2024 Sep 10;15:1454554. doi: 10.3389/fpls.2024.1454554 (PMC11423357; doi:10.3389/fpls.2024.1454554)
Supplement: Supplementary file 1 [file DataSheet1.pdf]

Supplementary Material

Systemic delivery of engineered compact AsCas12f by a positive-strand RNA virus vector enables highly efficient targeted mutagenesis in plants

Kazuhiro Ishibashi, Satoru Sukegawa, Masaki Endo, Naho Hara,

Osamu Nureki, Hiroaki Saika, Seiichi Toki

WT	attgtTTGTCACATGGAAATGCTATGGTtctagatgcaatagaatgatacatgcc	
#8	attgtTTGTCACATGGAAATGC-------------------gaatgatacatgcc	-19bp
	attgtTTGTCACATGGAAATGC---------------aatagaatgatacatgcc	-15bp
#9	attgtTTGTCACATG---------------------caatagaatgatacatgcc	-21bp
	attgtTTGTCACATGGAAA-------------gatgcaatagaatgatacatgcc	-13bp
#14	attgtTTGTCACATGGAAA--------------atgcaatagaatgatacatgcc	-14bp
	attgtTTGTCACATGGAAA--------------atgcaatagaatgatacatgcc	-14bp
#16	attgtTTGTCACATGGAAATGC-----------atgcaatagaatgatacatgcc	-11bp
	attgtTTGTCACATGGA------------------------------tacatgcc	-30bp
#17	attgtTTGTCACATGGAA--------------------------tgatacatgcc	-26bp
	attgtTTGTCACATGGAAATGCTA--------gatgcaatagaatgatacatgcc	-8bp
#20	attgtTTGTCACATGGAA---------------------tagaatgatacatgcc	-21bp
	attgtTTGTCACATGGAAATGC-----------atgcaatagaatgatacatgcc	-11bp

Supplementary Figure S1. Sequencing results on the *OsTubA3* site resulting from an engineered AsCas12f YKRA variant.

Sanger sequencing results from regenerated plants. Purple and red letters represent the PAM sequence and the target sequence, respectively. Underlined letters indicate microhomology sequences located at the breakpoint.

WT	TTGGTAGTAGCGACTCCATGGGGCATAAGTTAAGGATTCGT	
L3	TTGGTAGTAGCGACTCCATGGGGCA--AGTTAAGGATTCGT	-2 bp
	TTGGTAGTAGCGACTCCA---------AGTTAAGGATTCGT	-9 bp
	TTGGTAGTAGCGAC------------AAGTTAAGGATTCGT	-12 bp
	TTGGTAGTAGCGA-------------AAGTTAAGGATTCGT	-13 bp
	TTGGTAGTAGCGA--------------AGTTAAGGATTCGT	-14 bp
	TTGGTAGTAGCGAC----------ATAATTTAAGGATTCGT	-10 bp
	TTGGTAGTAGCGAC----------ATAAGTTAAGGATTCGT	-10 bp
	TTGGTAGTAGC---------------AAGTTAAGGATTCGT	-15 bp
	TTGGTAGTAGCGACTC-----------------GGATTCGT	-17 bp
	TTGGTAGTAGCGACTCA------------------ATTCGT	-18 bp

Supplementary Figure S2. Sequencing results on the *NbPDSa* site of the third upper leaves of pPZPVX-AsE10-NbPDS-inoculated *N. benthamiana* plants.

Purple and red letters represent the PAM sequence and the target sequence, respectively.

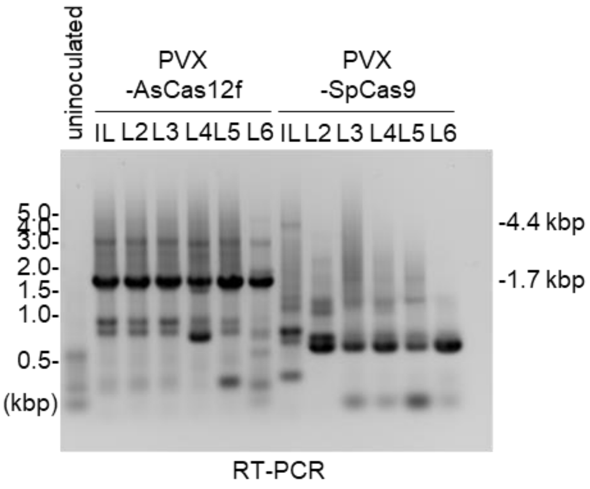

Supplementary Figure S3. RT-PCR analysis of the stability of inserted AsCas12f or SpCas9 sequences in the PVX vector

The leaf number (Lx) from the inoculated leaves (IL) is indicated. Expected lengths of SpCas9 (4.4 kbp)- and AsCas12f (1.7 kbp)-containing bands are indicated to the right.

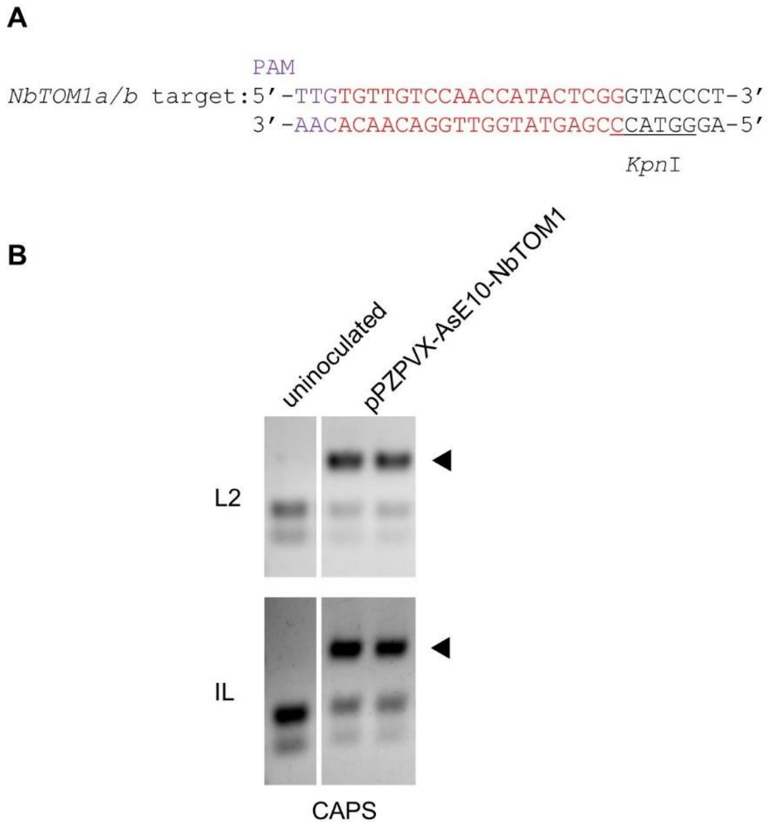

Supplementary Figure S4. Genome editing at the *NbTOM1* locus by PVX vector expressing engineered AsCas12f.

(A) Target sequence of AsCas12f in *NbTOM1*. Purple and red letters represent the PAM sequence and the target sequence, respectively. (B) Detection of targeted mutations in pPZPVX-AsE10-NbTOM1 inoculated *N. benthamiana* plants by CAPS at 6 dpi (lower panel) and 10 dpi (upper panel) for inoculated (IL) and the second upper leaves (L2), respectively. Each lane represents an independent plant. Similar results were obtained in two independent experiments, each involving inoculation of two plants.

Supplementary Table S1. Primers used in this study.

	Sequence (5'-3')
HMA for *OsTubA3* (Table 1)<br>PCR in regenerated plants (Figure S1)	CTGGATCTGACCTCGGGATGCTGTTA
	GCGGCGTGCTTTGGAAATGTTAAGTA
HMA for *OsDL* (Table 1)	CCGACCCTTCCATAAAATAGTG
	TTTGATACTACTTATTTGTGTTCAAGC
CAPS for *NbPDS* (Figure 2C)	TTAGGTTCACAAGTGGGACAATCTTC
	CAGCATCACACTTTCGCATTCAAAAC
Fragment analysis of regenerated shoots for *NbPDS* (Figure 2E)	5'-HEX-GCTTGCAAAGGAATTTGTTATGTTTTGG
	TTTAAAGGATTAAAGTCCTTTGTCAATCTTC
RT-PCR for PVX vector (Figure S3)	TTGCCGATCTCAAGCCACTCTCCGT
	GTAGTTGACCCTATGGGCTGTGTTG
CAPS for *NbTOM1* (Figure S4)	GACTGTATCCTCAAAGCTTTGATC
	CCTTAGGATGGAAGAGAAACACT

Supplementary Table S2. Vector sequences used in this study.

(A) T-DNA region of wild-type AsCas12f and sgRNA for *OsTubA3* expression vector.

GTTTACCCGCCAATATATCCTGTCAAACACTGATAGTTTAAACTGAAGGCGGGAAACGACAATCTGATCCTGGCGAAAGGG
GGATGTGCTGCAAGGCGATTAAGTTGGGTAACGCCAGGGTTTTCCCAGTCACGACGTTGTAAAACGACGGCCAGTGCCAAG
CTCTTAATTAAGGATCATGAACCAACGGCCTGGCTGTATTTGGTGGTTGTGTAGGGAGATGGGGAGAAGAAAAGCCCGATT
CTCTTCGCTGTGATGGGCTGGATGCATGCGGGGGAGCGGGAGGCCCAAGTACGTGCACGGTGAGCGGCCCACAGGGCGAGT
GTGAGCGCGAGAGGCGGGAGGAACAGTTTAGTACCACATTGCCCAGCTAACTCGAACGCGACCAACTTATAAACCCGCGCG
CTGTCGCTTGTGTTGATTCGTCGGTTCAGCGACGATAAGCCGAGAAGTGCCAATAAAACTGTTAAGTGGTTTGGTAACGCT
CGGTAAGGTCCGAAAGGAGAACCACTGAACTCACATGGAAATGCTATGGTTTTTTTGTCCCTTCGAAGGGCAATTGGCGC
GCCAAGCTTTAGAGATAATGAGCATTGCATGTCTAAGTTATAAAAAATTACCACATATTTTTTTTGTCACACTTGTTTGAA
GTGCAGTTTATCTATCTTTATACATATATTTAAACTTTACTCTACGAATAATATAATCTATAGTACTACAATAATATCAGT
GTTTTAGAGAATCATATAAATGAACAGTTAGACATGGTCTAAAGGACAATTGAGTATTTTGACAACAGGACTCTACAGTTT
TATCTTTTTAGTGTGCATGTGTTCTCCTTTTTTTTTGCAAATAGCTTCACCTATATAATACTTCATCCATTTTATTAGTAC
ATCCATTTAGGGTTTAGGGTTAATGGTTTTTATAGACTAATTTTTTTAGTACATCTATTTTATTCTATTTTAGCCTCTAAA
TTAAGAAAACTAAAACTCTATTTTAGTTTTTTTATTTAATAATTTAGATATAAAATAGAATAAAATAAAGTGACTAAAAAT
TAAACAAATACCCTTTAAGAAATTAAAAAAACTAAGGAAACATTTTTCTTGTTTCGAGTAGATAATGCCAGCCTGTTAAAC
GCCGTCGACGAGTCTAACGGACACCAACCAGCGAACCAGCAGCGTCGCGTCGGGCCAAGCGAAGCAGACGGCACGGCATCT
CTGTCGCTGCCTCTGGACCCCTCTCGAGAGTTCCGCTCCACCGTTGGACTTGCTCCGCTGTCGGCATCCAGAAATTGCGTG
GCGGAGCGGCAGACGTGAGCCGGCACGGCAGGCGGCCTCCTCCTCCTCTCACGGCACGGCAGCTACGGGGGATTCCTTTCC
CACCGCTCCTTCGCTTTCCCTTCCTCGCCCGCCGTAATAAATAGACACCCCCTCCACACCCTCTTTCCCCAACCTCGTGTT
GTTCGGAGCGCACACACACACACAACCAGATCTCCCCCAAATCCACCCGTCGGCACCTCCGCTTCAAGGTACGCCGCTCGTCC
TCCCCCCCCCCCCCTCTCTACCTTCTCTAGATCGGCGTTCCGGTCCATGGTTAGGGCCCGGTAGTTCTACTTCTGTTCATG
TTTGTGTTAGATCCGTGTTTGTGTTAGATCCGTGCTGCTAGCGTTCGTACACGGATGCGACCTGTACGTCAGACACGTTCT
GATTGCTAACTTGCCAGTGTTTCTCTTTGGGGAATCCTGGGATGGCTCTAGCCGTTCCGCAGACGGGATCGATTTCATGAT
TTTTTTTGTTTCGTTGCATAGGGTTTGGTTTGCCCTTTTCCTTTATTTCAATATATGCCGTGCACTTGTTTGTCGGGTCAT
CTTTTCATGCTTTTTTTTGTCTTGGTTGTGATGATGTGGTCTGGTTGGGCGGTCGTTCTAGATCGGAGTAGAATTCTGTTT
CAAACTACCTGGTGGATTTATTAATTTTGGATCTGTATGTGTGTGCCATACATATTCATAGTTACGAATTGAAGATGATGG
ATGGAAATATCGATCTAGGATAGGTATACATGTTGATGCGGGTTTTACTGATGCATATACAGAGATGCTTTTTGTTCGCTT
GGTTGTGATGATGTGGTGTGGTTGGGCGGTCGTTCATTCGTTCTAGATCGGAGTAGAATACTGTTTCAAACTACCTGGTGT
ATTTATTAATTTTGGAACTGTATGTGTGTGTCATACATCTTCATAGTTACGAGTTTAAGATGGATGGAAATATCGATCTAG
GATAGGTATACATGTTGATGTGGGTTTTACTGATGCATATACATGATGGCATATGCAGCATCTATTCATATGCTCTAACCT
TGAGTACCTATCTATTATAATAAACAAGTATGTTTTATAATTATTTTGATCTTGATATACTTGGATGATGGCATATGCAGC
AGCTATATGTGGATTTTTTTAGCCCTGCCTTCATACGCTATTTATTTGCTTGGTACTGTTTCTTTTGTCGATGCTCACCCT
GTTGTTTGGTGTTACTTCTGCAGGAATTCCAAGCAACGAACTGCGAGTGATTCAAGAAAAAAGAAAACCTGAGCTTTCGAT
CTCTACGGAGTGGTTTCTTGTTCTTTGAAAAAGAGGGGGATTAGTCGACATGATCAAGGTGTACCGCTACGAGATCGTGAA
GCCGCTCGACCTCGACTGGAAAGAGTTCGGCACCATCCTCCGCCAGCTCCAGCAAGAGACAAGGTTCGCCCTCAACAAGGC
CACACAACTCGCCTGGGAGTGGATGGGCTTCTCCAGCGACTACAAGGACAACCACGGCGAGTACCCGAAGTCCAAGGACAT
CCTCGGCTACACCAACGTGCACGGCTACGCCTACCACACCATCAAGACCAAGGCCTACAGGCTCAACTCCGGCAACCTCAG
CCAGACGATCAAGAGGGCCACCGACAGGTTCAAGGCGTACCAGAAAGAGATCCTCCGCGGCGACATGAGCATCCCGTCCTA
CAAGAGGGACTACCCGCTGGACCTCATCAAAGAGAACATCTCCGTCAACCGCATGAACCATGGCGACTATATCGCGAGCCT
CAGCCTCCTCAGCAACCCGGCTAAGCAAGAGATGAACGTCAAGCGCAAGATCTCCGTGATCATCATCGTTCGCGGCGCTGG
CAAGACCATCATGGACAGAATTCTCTCCGGCGAGTATCAGGTGTCCGCCAGCCAGATCATCCACAAGGACCGCAAGAACAA
GTGGTATCTCAACATCTCCTACAGGTTCGAGCCGCAGACCAGGGTCCTCGACCTGAACAAGATCATGGGCATCGATCTCGG
CGTGGCCGTGGCCGCTTACATGGCCTTCCAACATACCCCGGCCAGGTACAAGCTTGAAGGCGGCGAGATCGAGAACTTCCG
CAGGCAAGTTGAGTCCCGCCGCATCTCTATGCTCCGCCAAGGCAAATACGCCGGTGGCGCTAGAGGCGGACATGGCAGGGA
TAAGAGGATCAAGCCGATCGAGCAGCTCCGCGACAAGATCGCCAACTTCAGGGACACCACCAACCACCGCTACTCCCGCTA
CATCGTGGACATGGCCATCAAAGAAGGCTGCGGCACGATCCAGATGGAAGATCTCACCAACATCCGCGACATCGGCTCCCG
GTTCCTCCAGAACTGGACCTACTACGACCTCCAGCAGAAGATCATCTACAAGGCCGAAGAGGCCGGCATCAAGGTGATCAA
GATCGACCCGCAGTACACCAGCCAGAGGTGCTCTGAGTGCGGCAACATCGATAGCGGCAACAGGATCGGCCAGGCCATCTT
TAAGTGCCGCGCTTGCGGCTACGAGGCCAACGCCGATTATAACGCCGCGAGGAATATCGCGATCCCGAACATCGACAAGAT
TATCGCCGAGTCCATCAAGGGTACCGGAGGCTCTAAGAGGACCGCTGATGGCAGCGAGTTTGAGCCAAAGAAGAAGAGGAA
GGTTTGAGAGCTCGATCTGTCGATCGACAAGCTCGAGTTTCTCCATAATAATGTGTGAGTAGTTCCCAGATAAGGGAATTA
GGGTTCCTATAGGGTTTCGCTCATGTGTTGAGCATATAAGAAACCCTTAGTATGTATTTGTATTTGTAAAATACTTCTATC
AATAAAATTTCTAATTCCTAAAACCAAAATCCAGTACTAAAATCCAGATCCCCCGAAGAATTTCCCCGATCGTTCAAACAT
TTGGCAATAAAGTTTCTTAAGATTGAATCCTGTTGCCGGTCTTGCGATGATTATCATATAATTTCTGTTGAATTACGTTAA
GCATGTAATAATTAACATGTAATGCATGACGTTATTTATGAGATGGGTTTTTATGATTAGAGTCCCGCAATTATACATTTA

ATACGCGATAGAAAACAAAATATAGCGCGCAAACTAGGATAAATTATCGCGCGCGGTGTCATCTATGTTACTAGATCGGAA
TTCGTAATCATGGTCATAGCTGTTTCCTGTGGGATCCGTCCCCAGATTAGCCTTTTCAATTTCAGAAAGAATGCTAACCCA
CAGATGGTTAGAGAGGCTTACGCAGCAGGTCTCATCAAGACGATCTACCCGAGCAATAATCTCCAGGAAATCAAATACCTT
CCCAAGAAGGTTAAAGATGCAGTCAAAAGATTCAGGACTAACTGCATCAAGAACACAGAGAAAGATATATTTCTCAAGATC
AGAAGTACTATTCCAGTATGGACGATTCAAGGCTTGCTTCACAAACCAAGGCAAGTAATAGAGATTGGAGTCTCTAAAAAG
GTAGTTCCCACTGAATCAAAGGCCATGGAGTCAAAGATTCAAATAGAGGACCTAACAGAACTCGCCGTAAAGACTGGCGAA
CAGTTCATACAGAGTCTCTTACGACTCAATGACAAGAAGAAAATCTTCGTCAACATGGTGGAGCACGACACACTTGTCTAC
TCCAAAAATATCAAAGATACAGTCTCAGAAGACCAAAGGGCAATTGAGACTTTTCAACAAAGGGTAATATCCGGAAACCTC
CTCGGATTCCATTGCCCAGCTATCTGTCACTTTATTGTGAAGATAGTGGAAAAGGAAGGTGGCTCCTACAAATGCCATCAT
TGCGATAAAGGAAAGGCCATCGTTGAAGATGCCTCTGCCGACAGTGGTCCCAAAGATGGACCCCCACCCACGAGGAGCATC
GTGGAAAAAGAAGACGTTCCAACCACGTCTTCAAAGCAAGTGGATTGATGTGATATCTCCACTGACGTAAGGGATGACGCA
CAATCCCACTATCCTTCGCAAGACCCTTCCTCTATATAAGGAAGTTCATTTCATTTGGAGAGAACACGGGGGAACTAGTAT
GAAAAAGCCTGAACTCACCGCGACGTCTGTCGAGAAGTTTCTGATCGAAAAGTTCGACAGCGTCTCCGACCTGATGCAGCT
CTCGGAGGGCGAAGAATCTCGTGCTTTCAGCTTCGATGTAGGAGGGCGTGGATATGTCCTGCGGGTAAATAGCTGCGCCGA
TGGTTTCTACAAAGATCGTTATGTTTATCGGCACTTTGCATCGGCCGCGCTCCCGATTCCGGAAGTGCTTGACATTGGGGA
GTTTAGCGAGAGCCTGACCTATTGCATCTCCCGCCGTTCACAGGGTGTCACGTTGCAAGACCTGCCTGAAACCGAACTGCC
CGCTGTTCTACAACCGGTCGCGGAGGCTATGGATGCGATCGCTGCGGCCGATCTTAGCCAGACGAGCGGGTTCGGCCCATT
CGGACCGCAAGGAATCGGTCAATACACTACATGGCGTGATTTtATATGCGCGATTGCTGATCCCCATGTGTATCACTGGCA
AACTGTGATGGACGACACCGTCAGTGCGTCCGTCGCGCAGGCTCTCGATGAGCTGATGCTTTGGGCCGAGGACTGCCCCGA
AGTCCGGCACCTCGTGCACGCGGATTTCGGCTCCAACAATGTCCTGACGGACAATGGCCGCATAACAGCGGTCATTGACTG
GAGCGAGGCGATGTTCGGGGATTCCCAATACGAGGTCGCCAACATCTTCTTCTGGAGGCCGTGGTTGGCTTGTATGGAGCA
GCAGACGCGCTACTTCGAGCGGAGGCATCCGGAGCTTGCAGGATCGCCACGACTCCGGGCGTATATGCTCCGCATTGGTCT
TGACCAACTCTATCAGAGCTTGGTTGACGGCAATTTCGATGATGCAGCTTGGGCGCAGGGTCGATGCGACGCAATCGTCCG
ATCCGGAGCCGGGACTGTCGGGCGTACACAAATCGCCCGCAGAAGCGCGGCCGTCTGGACCGATGGCTGTGTAGAAGTACT
CGCCGATAGTGGAAACCGACGCCCCAGCACTCGTCCGAGGGCAAAGAAATAGGGGCCCCGATCGTTCAAACATTTGGCAAT
AAAGTTTCTTAAGATTGAATCCTGTTGCCGGTCTTGCGATGATTATCATATAATTTCTGTTGAATTACGTTAAGCATGTAA
TAATTAACATGTAATGCATGACGTTATTTATGAGATGGGTTTTTATGATTAGAGTCCCGCAATTATACATTTAATACGCGA
TAGAAAACAAAATATAGCGCGCAAACTAGGATAAATTATCGCGCGCGGTGTCATCTATGTTACTAGATCAATTCAATTCGG
CGTTAATTCAGTACATTAAAAACGTCCGCAATGTGTTATTAAGTTGTCTAAGCGTCAATTTGTTTACACCACAATATATCC
TGCCA

RB, OsU6-2 promoter, sgRNA for AsCas12f (ΔS3-5_v7), *OsTubA3* target sequence, poly T, maize ubiqutin1 promoter, 5' untranslated region of rice alcohol dehydrogenase, Rice codon-optimized wild-type AsCas12f, linker, SV40 NLS, 35S terminator, NOS terminator, 35S promoter, hygromycin phosphotransferase, LB

(B) Sequence of AsCas12f variants for rice transformation vectors. Mutations are highlighted in red.

(a) YHAM

ATGATCAAGGTGTACCGCTACGAGATCGTGAAGCCGCTCGACCTCGACTGGAAAGAGTTCGGCACCATCCTCCGCCAGCTC
CAGCAAGAGACAAGGTTCGCCCTCAACAAGGCCACACAACTCGCCTGGGAGTGGATGGGCTaCTCCAGCGACTACAAGGAC
AACCACGGCGAGTACCCGAAGTCCAAGGACATCCTCGGCTACACCAACGTGCACGGCTACGCCTACCACACCATCAAGACC
AAGGCCTACAGGCTCAACTCCGGCAACCTCAGCCAGACGATCAAGAGGGCCACCGACAGGTTCAAGGCGTACCAGAAAGAG
ATCCTCCGCGGCGACATGAGCATCCCGTCCTACAAGAGGGACATCCCGCTGGACCTCATCAAAGAGAACATCTCCGTCAAC
CGCATGAACCATGGCGACTATATCGCGAGCCTCAGCCTCCTCAGCAACCCGGCTAAGCAAGAGATGAACGTCAAGCGCAAG
ATCTCCGTGATCATCATCGTTCGCGGCGCTGGCAAGACCATCATGGACAGAATTCTCTCCGGCGAGTATCAGGTGcaCGCC
AGCCAGATCATCCACGACGACCGCAAGAACAAGTGGTATCTCAACATCTCCTACGACTTCGAGCCGCAGACCAGGGTCCTC
GACCTGAACAAGATCATGGGCATCGATCTCGGCGTGGCCGTGGCCGcTTACATGGCCTTCCAACATACCCCGGCCAGGTAC
AAGCTTGAAGGCGGCGAGATCGAGAACTTCCGCAGGCAAGTTGAGTCCCGCCGCATCTCTATGCTCCGCCAAGGCAAATAC
GCCGGTGGCGCTAGAGGCGGACATGGCAGGGATAAGAGGATCAAGCCGATCGAGCAGCTCCGCGACAAGATCGCCAACTTC
AGGGACACCACCAACCACCGCTACTCCCGCTACATCGTGGACATGGCCATCAAAatgGGCTGCGGCACGATCCAGATGGAA

GATCTCACCAACATCCGCGACATCGGCTCCCGGTTCCTCCAGAACTGGACCTACTACGACCTCCAGCAGAAGATCATCTAC
AAGGCCGAAGAGGCCGGCATCAAGGTGATCAAGATCGACCCGCAGTACACCAGCCAGAGGTGCTCTGAGTGCGGCAACATC
GATAGCGGCAACAGGATCGGCCAGGCCATCTTTAAGTGCCGCGCTTGCGGCTACGAGGCCAACGCCGATTATAACGCCGCG
AGGAATATCGCGATCCCGAACATCGACAAGATTATCGCCGAGTCCATCAAG

(b) HAMN

ATGATCAAGGTGTACCGCTACGAGATCGTGAAGCCGCTCGACCTCGACTGGAAAGAGTTCGGCACCATCCTCCGCCAGCTC
CAGCAAGAGACAAGGTTCGCCCTCAACAAGGCCACACAACTCGCCTGGGAGTGGATGGGCTTCTCCAGCGACTACAAGGAC
AACCACGGCGAGTACCCGAAGTCCAAGGACATCCTCGGCTACACCAACGTGCACGGCTACGCCTACCACACCATCAAGACC
AAGGCCTACAGGCTCAACTCCGGCAACCTCAGCCAGACGATCAAGAGGGCCACCGACAGGTTCAAGGCGTACCAGAAAGAG
ATCCTCCGCGGCGACATGAGCATCCCGTCCTACAAGAGGGACATCCCGCTGGACCTCATCAAAGAGAACATCTCCGTCAAC
CGCATGAACCATGGCGACTATATCGCGAGCCTCAGCCTCCTCAGCAACCCGGCTAAGCAAGAGATGAACGTCAAGCGCAAG
ATCTCCGTGATCATCATCGTTCGCGGCGCTGGCAAGACCATCATGGACAGAATTCTCTCCGGCGAGTATCAGGTGcaCGCC
AGCCAGATCATCCACGACGACCGCAAGAACAAGTGGTATCTCAACATCTCCTACGACTTCGAGCCGCAGACCAGGGTCCTC
GACCTGAACAAGATCATGGGCATCGATCTCGGCGTGGCCGTGGCCGcTTACATGGCCTTCCAACATACCCCGGCCAGGTAC
AAGCTTGAAGGCGGCGAGATCGAGAACTTCCGCAGGCAAGTTGAGTCCCGCCGCATCTCTATGCTCCGCCAAGGCAAATAC
GCCGGTGGCGCTAGAGGCGGACATGGCAGGGATAAGAGGATCAAGCCGATCGAGCAGCTCCGCGACAAGATCGCCAACTTC
AGGGACACCACCAACCACCGCTACTCCCGCTACATCGTGGACATGGCCATCAAAatgGGCTGCGGCACGATCCAGATGGAA
GATCTCACCAACATCCGCGACATCGGCTCCCGGTTCCTCCAGAACTGGACCTACTACGACCTCCAGCAGAAGATCATCTAC
AAGGCCGAAGAGGCCGGCATCAAGGTGATCAAGATCGACCCGCAGTACACCAGCCAGAGGTGCTCTGAGTGCGGCAACATC
GATAGCGGCAACAGGATCGGCCAGGCCAaCTTTAAGTGCCGCGCTTGCGGCTACGAGGCCAACGCCGATTATAACGCCGCG
AGGAATATCGCGATCCCGAACATCGACAAGATTATCGCCGAGTCCATCAAG

(c) YKRA

ATGATCAAGGTGTACCGCTACGAGATCGTGAAGCCGCTCGACCTCGACTGGAAAGAGTTCGGCACCATCCTCCGCCAGCTC
CAGCAAGAGACAAGGTTCGCCCTCAACAAGGCCACACAACTCGCCTGGGAGTGGATGGGCTTCTCCAGCGACTACAAGGAC
AACCACGGCGAGTACCCGAAGTCCAAGGACATCCTCGGCTACACCAACGTGCACGGCTACGCCTACCACACCATCAAGACC
AAGGCCTACAGGCTCAACTCCGGCAACCTCAGCCAGACGATCAAGAGGGCCACCGACAGGTTCAAGGCGTACCAGAAAGAG
ATCCTCCGCGGCGACATGAGCATCCCGTCCTACAAGAGGGACtaCCCGCTGGACCTCATCAAAGAGAACATCTCCGTCAAC
CGCATGAACCATGGCGACTATATCGCGAGCCTCAGCCTCCTCAGCAACCCGGCTAAGCAAGAGATGAACGTCAAGCGCAAG
ATCTCCGTGATCATCATCGTTCGCGGCGCTGGCAAGACCATCATGGACAGAATTCTCTCCGGCGAGTATCAGGTGTCCGCC
AGCCAGATCATCCACaAgGACCGCAAGAACAAGTGGTATCTCAACATCTCCTACaggTTCGAGCCGCAGACCAGGGTCCTC
GACCTGAACAAGATCATGGGCATCGATCTCGGCGTGGCCGTGGCCGcTTACATGGCCTTCCAACATACCCCGGCCAGGTAC
AAGCTTGAAGGCGGCGAGATCGAGAACTTCCGCAGGCAAGTTGAGTCCCGCCGCATCTCTATGCTCCGCCAAGGCAAATAC
GCCGGTGGCGCTAGAGGCGGACATGGCAGGGATAAGAGGATCAAGCCGATCGAGCAGCTCCGCGACAAGATCGCCAACTTC
AGGGACACCACCAACCACCGCTACTCCCGCTACATCGTGGACATGGCCATCAAAGAAGGCTGCGGCACGATCCAGATGGAA
GATCTCACCAACATCCGCGACATCGGCTCCCGGTTCCTCCAGAACTGGACCTACTACGACCTCCAGCAGAAGATCATCTAC
AAGGCCGAAGAGGCCGGCATCAAGGTGATCAAGATCGACCCGCAGTACACCAGCCAGAGGTGCTCTGAGTGCGGCAACATC
GATAGCGGCAACAGGATCGGCCAGGCCATCTTTAAGTGCCGCGCTTGCGGCTACGAGGCCAACGCCGATTATAACGCCGCG
AGGAATATCGCGATCCCGAACATCGACAAGATTATCGCCGAGTCCATCAAG

(d) HKRA

ATGATCAAGGTGTACCGCTACGAGATCGTGAAGCCGCTCGACCTCGACTGGAAAGAGTTCGGCACCATCCTCCGCCAGCTC
CAGCAAGAGACAAGGTTCGCCCTCAACAAGGCCACACAACTCGCCTGGGAGTGGATGGGCTTCTCCAGCGACTACAAGGAC
AACCACGGCGAGTACCCGAAGTCCAAGGACATCCTCGGCTACACCAACGTGCACGGCTACGCCTACCACACCATCAAGACC
AAGGCCTACAGGCTCAACTCCGGCAACCTCAGCCAGACGATCAAGAGGGCCACCGACAGGTTCAAGGCGTACCAGAAAGAG
ATCCTCCGCGGCGACATGAGCATCCCGTCCTACAAGAGGGACcaCCCGCTGGACCTCATCAAAGAGAACATCTCCGTCAAC
CGCATGAACCATGGCGACTATATCGCGAGCCTCAGCCTCCTCAGCAACCCGGCTAAGCAAGAGATGAACGTCAAGCGCAAG
ATCTCCGTGATCATCATCGTTCGCGGCGCTGGCAAGACCATCATGGACAGAATTCTCTCCGGCGAGTATCAGGTGTCCGCC
AGCCAGATCATCCACaAgGACCGCAAGAACAAGTGGTATCTCAACATCTCCTACaggTTCGAGCCGCAGACCAGGGTCCTC
GACCTGAACAAGATCATGGGCATCGATCTCGGCGTGGCCGTGGCCGcTTACATGGCCTTCCAACATACCCCGGCCAGGTAC
AAGCTTGAAGGCGGCGAGATCGAGAACTTCCGCAGGCAAGTTGAGTCCCGCCGCATCTCTATGCTCCGCCAAGGCAAATAC
GCCGGTGGCGCTAGAGGCGGACATGGCAGGGATAAGAGGATCAAGCCGATCGAGCAGCTCCGCGACAAGATCGCCAACTTC
AGGGACACCACCAACCACCGCTACTCCCGCTACATCGTGGACATGGCCATCAAAGAAGGCTGCGGCACGATCCAGATGGAA
GATCTCACCAACATCCGCGACATCGGCTCCCGGTTCCTCCAGAACTGGACCTACTACGACCTCCAGCAGAAGATCATCTAC

AAGGCCGAAGAGGCCGGCATCAAGGTGATCAAGATCGACCCGCAGTACACCAGCCAGAGGTGCTCTGAGTGCGGCAACATC
GATAGCGGCAACAGGATCGGCCAGGCCATCTTTAAGTGCCGCGCTTGCGGCTACGAGGCCAACGCCGATTATAACGCCGCG
AGGAATATCGCGATCCCGAACATCGACAAGATTATCGCCGAGTCCATCAAG

(e) MKRA

ATGATCAAGGTGTACCGCTACGAGATCGTGAAGCCGCTCGACCTCGACTGGAAAGAGTTCGGCACCATCCTCCGCCAGCTC
CAGCAAGAGACAAGGTTCGCCCTCAACAAGGCCACACAACTCGCCTGGGAGTGGATGGGCTTCTCCAGCGACTACAAGGAC
AACCACGGCGAGTACCCGAAGTCCAAGGACATCCTCGGCTACACCAACGTGCACGGCTACGCCTACCACACCATCAAGACC
AAGGCCTACAGGCTCAACTCCGGCAACCTCAGCatGACGATCAAGAGGGCCACCGACAGGTTCAAGGCGTACCAGAAAGAG
ATCCTCCGCGGCGACATGAGCATCCCGTCCTACAAGAGGGACATCCCGCTGGACCTCATCAAAGAGAACATCTCCGTCAAC
CGCATGAACCATGGCGACTATATCGCGAGCCTCAGCCTCCTCAGCAACCCGGCTAAGCAAGAGATGAACGTCAAGCGCAAG
ATCTCCGTGATCATCATCGTTCGCGGCGCTGGCAAGACCATCATGGACAGAATTCTCTCCGGCGAGTATCAGGTGTCCGCC
AGCCAGATCATCCACaAgGACCGCAAGAACAAGTGGTATCTCAACATCTCCTACaggTTCGAGCCGCAGACCAGGGTCCTC
GACCTGAACAAGATCATGGGCATCGATCTCGGCGTGGCCGTGGCCGcTTACATGGCCTTCCAACATACCCCGGCCAGGTAC
AAGCTTGAAGGCGGCGAGATCGAGAACTTCCGCAGGCAAGTTGAGTCCCGCCGCATCTCTATGCTCCGCCAAGGCAAATAC
GCCGGTGGCGCTAGAGGCGGACATGGCAGGGATAAGAGGATCAAGCCGATCGAGCAGCTCCGCGACAAGATCGCCAACTTC
AGGGACACCACCAACCACCGCTACTCCCGCTACATCGTGGACATGGCCATCAAAGAAGGCTGCGGCACGATCCAGATGGAA
GATCTCACCAACATCCGCGACATCGGCTCCCGGTTCCTCCAGAACTGGACCTACTACGACCTCCAGCAGAAGATCATCTAC
AAGGCCGAAGAGGCCGGCATCAAGGTGATCAAGATCGACCCGCAGTACACCAGCCAGAGGTGCTCTGAGTGCGGCAACATC
GATAGCGGCAACAGGATCGGCCAGGCCATCTTTAAGTGCCGCGCTTGCGGCTACGAGGCCAACGCCGATTATAACGCCGCG
AGGAATATCGCGATCCCGAACATCGACAAGATTATCGCCGAGTCCATCAAG

(f) enAsCas12f

ATGATCAAGGTGTACCGCTACGAGATCGTGAAGCCGCTCGACCTCGACTGGAAAGAGTTCGGCACCATCCTCCGCCAGCTC
CAGCAAGAGACAAGGTTCGCCCTCAACAAGGCCACACAACTCGCCTGGGAGTGGATGGGCTTCTCCAGCGACTACAAGGAC
AACCACGGCGAGTACCCGAAGTCCAAGGACATCCTCGGCTACACCAACGTGCACGGCTACGCCTACCACACCATCAAGACC
AAGGCCTACAGGCTCAACTCCGGCAACCTCAGCCAGACGATCAAGAGGGCCACCGACAGGTTCAAGGCGTACCAGAAAGAG
ATCCTCCGCGGCGACATGAGCATCCCGTCCTACAAGAGGGACATCCCGCTGGACCTCATCAAAGAGAACATCTCCGTCAAC
CGCATGAACCATGGCGACTATATCGCGAGCCTCAGCCTCCTCAGCAACCCGGCTAAGCAAGAGATGAACGTCAAGCGCAAG
ATCTCCGTGATCATCATCGTTCGCGGCGCTGGCAAGACCATCATGGACAGAATTCTCTCCGGCGAGTATCAGGTGTCCGCC
AGCCAGATCATCCACGACaAgCGCAAGAAgAAGTGGTATCTCAACATCTCCTACGACTTCGAGCCGCAGACCAGGGTCCTC
GACCTGAACAAGATCATGGGCATCGATCTCGGCGTGGCCGTGGCCGTTTACATGGCCTTCCAACATACCCCGGCCAGGTAC
AAGCTTGAAGGCGGCGAGATCGAGAACTTCCGCAGGCAAGTTGAGTCCCGCCGCATCTCTATGCTCCGCCAAGGCAAATAC
GCCGGTGGCGCTAGAaGgGGACATGGCAGGGATAAGAGGATCAAGCCGATCGAGCAGCTCCGCGACAAGATCGCCAACTTC
AGGGACACCACCAACCACCGCTACTCCCGCTACATCGTGGACATGGCCATCAAAGAAGGCTGCGGCACGATCCAGATGGAA
GATCTCACCggCATCCGCGACATCGGCTCCCGGTTCCTCCAGAACTGGACCTACTACGACCTCCAGCAGAAGATCATCTAC
AAGGCCGAAGAGGCCGGCATCAAGGTGATCAAGATCaggCCGCAGTACACCAGCCAGAGGTGCTCTGAGTGCGGCAACATC
GATAGCGGCAACAGGATCGGCCAGGCCATCTTTAAGTGCCGCGCTTGCGGCTACGAGGCCAACGCCGATTATAACGCCGCG
AGGAATATCGCGATCCCGAACATCGACAAGATTATCGCCGAGTCCATCAAG

(C) T-DNA region of the PVX vector for AsCas12f and sgRNA for *NbPDS* expression (pPZPVX-AsE10-NbPDS).

TGGCAGGATATATTGTGGTGTAAACAAATTGACGCTTAGACAACTTAATAACACATTGCGGACGTTTTTAATGTACTGAAT
TAACGCCGAATTGCTCTAGCATTCGCCATTCAGGCTGCGCAACTGTTGGGAAGGGCGATCGGTGCGGGCCTCTTCGCTATT
ACGCCAGCTGGCGAAAGGGGGATGTGCTGCAAGGCGATTAAGTTGGGTAACGCCAGGGTTTTCCCAGTCACGACGTTGTAA
AACGACGGCCAGTGCCAAGCTGGCGCGTCTCAGAAGACCAAAGGGCAATTGAGACTTTTCAACAAAGGGTAATATCCGGAA
ACCTCCTCGGATTCCATTGCCCAGCTATCTGTCACTTTATTGTGAAGATAGTGGAAAAGGAAGGTGGCTCCTACAAATGCC
ATCATTGCGATAAAGGAAAGGCCATCGTTGAAGATGCCTCTGCCGACAGTGGTCCCAAAGATGGACCCCCACCCACGAGGA
GCATCGTGGAAAAAGAAGACGTTCCAACCACGTCTTCAAAGCAAGTGGATTGATGTGATATCTCCACTGACGTAAGGGATG
ACGCACAATCCCACTATCCTTCGCAAGACCCTTCCTCTATATAAGGAAGTTCATTTCATTTGGAGAGGAGAAAACTAAACC
ATACACCACCAACACAACCAAACCCACCACGCCCAATTGTTACACACCCGCTTGAAAAAGAAAGTTTAACAAATGGCCAAG

GTGCGCGAGGTTTACCAATCTTTTACAGACTCCACCACAAAAACTCTCATCCAAGATGAGGCTTATAGAAACATTCGCCCC
ATCATGGAAAAACACAAACTAGCTAACCCTTACGCTCAAACGGTTGAAGCGGCTAATGATCTAGAGGGGTTCGGCATAGCC
ACCAATCCCTATAGCATTGAATTGCATACACATGCAGCCGCTAAGACCATAGAGAATAAACTTCTAGAGGTGCTTGGTTCC
ATCCTACCACAAGAACCTGTTACATTTATGTTTCTTAAACCCAGAAAGCTAAACTACATGAGAAGAAACCCGCGGATCAAG
GACATTTTCCAAAATGTTGCCATTGAACCAAGAGACGTAGCCAGGTACCCCAAGGAAACAATAATTGACAAACTCACAGAG
ATCACAACGGAAACAGCATACATTAGTGACACTCTGCACTTCTTGGATCCGAGCTACATAGTGGAGACATTCCAAAACTGC
CCAAAATTGCAAACATTGTATGCGACCTTAGTTCTCCCCGTTGAGGCAGCCTTTAAAATGGAAAGCACTCACCCGAACATA
TACAGCCTCAAATACTTCGGAGATGGTTTCCAGTATATACCAGGCAACCATGGTGGCGGGGCATACCATCATGAATTCGCT
CATCTACAATGGCTCAAAGTGGGAAAGATCAAGTGGAGGGACCCCAAGGATAGCTTTCTCGGACATCTCAATTACACGACT
GAGCAGGTTGAGATGCACACAGTGACAGTACAGTTGCAGGAATCGTTCGCGGCAAACCACTTGTACTGCATCAGGAGAGGA
GACTTGCTCACACCGGAGGTGCGCACTTTCGGCCAACCTGACAGGTACGTGATTCCACCACAGATCTTCCTCCCAAAAGTT
CACAACTGCAAGAAGCCGATTCTCAAGAAAACTATGATGCAGCTCTTCTTGTATGTTAGGACAGTCAAGGTCGCAAAAAAT
TGTGACATTTTTGCCAAAGTCAGACAATTAATTAAATCATCTGACTTGGACAAATACTCTGCTGTGGAACTGGTTTACTTA
GTAAGCTACATGGAGTTCCTTGCCGATTTACAAGCTACCACCTGCTTCTCAGACACACTTTCTGGTGGCTTGCTAACAAAG
ACCCTTGCACCGGTGAGGGCTTGGATACAAGAGAAAAAGATGCAGCTGTTTGGTCTTGAGGACTACGCGAAGTTAGTCAAA
GCAGTTGATTTCCACCCGGTGGATTTTTCTTTCAAAGTGGAAACTTGGGACTTCAGATTCCACCCCTTGCAAGCGTGGAAA
GCCTTCCGACCAAGGGAAGTGTCGGATGTAGAGGAAATGGAAAGTTTGTTCTCAGATGGGGACCTGCTTGATTGCTTCACA
AGAATGCCAGCTTATGCGGTAAACGCAGAGGAAGATTTAGCTGCAATCAGGAAAACGCCCGAGATGGATGTCGGTCAAGAA
GTTAAAGAGCCTGCAGGAGACAGAAATCAATACTCAAACCCTGCAGAAACTTTCCTCAACAAGCTCCACAGGAAACACAGT
AGGGAGGTGAAACACCAGGCCGCAAAGAAAGCTAAACGCCTAGCTGAAATCCAGGAGTCAATGAGAGCTGAAGGTGATGCC
GAACCAAATGAAATAAGCGGGACGATGGGGGCAATACCCAGCAACGCCGAACTTCCTGGCACGAATGATGCCAGACAAGAA
CTCACACTCCCAACCACTAAACCTGTCCCTGCAAGGTGGGAAGATGCTTCATTCACAGATTCTAGTGTGGAAGAGGAGCAG
GTTAAACTCCTTGGAAAAGAAACCGTTGAAACAGCGACGCAACAAGTCATCGAAGGACTTCCTTGGAAACACTGGATTCCT
CAATTAAATGCTGTTGGATTCAAGGCGCTGGAAATTCAGAGGGATAGGAGTGGAACAATGATCATGCCCATCACAGAAATG
GTCTCCGGGCTGGAAAAAGAGGACTTCCCTGAAGGAACTCCAAAAGAGTTGGCACGAGAATTGTTCGCTATGAACAGAAGC
CCTGCCACCATCCCTTTGGACCTGCTTAGAGCCAGAGACTACGGCAGTGATGTAAAGAACAAGAGAATTGGTGCCATCACA
AAGACACAGGCAACGAGTTGGGGCGAATACTTGACAGGAAAGATAGAAAGCTTAACTGAGAGGAAAGTTGCGACTTGTGTC
ATTCATGGAGCTGGAGGTTCTGGAAAAAGTCATGCCATCCAGAAGGCATTGAGAGAAATTGGCAAGGGCTCGGACATCACT
GTAGTCCTGCCGACCAATGAACTGCGGCTAGATTGGAGTAAGAAAGTGCCTAACACTGAGCCCTATATGTTCAAGACCTCT
GAAAAGGCGTTAATTGGGGGAACAGGCAGCATAGTCATCTTTGACGATTACTCAAAACTTCCTCCCGGTTACATAGAAGCC
TTAGTCTGTTTCTACTCTAAAATCAAGCTAATCATTCTAACAGGAGATAGCAGACAAAGCGTCTACCATGAAACTGCTGAG
GACGCCTCCATCAGGCATTTGGGACCAGCAACAGAGTACTTCTCAAAATACTGCCGATACTATCTCAATGCCACACACCGC
AACAAGAAAGATCTTGCGAACATGCTTGGTGTCTACAGTGAGAGAACGGGAGTCACCGAAATCAGCATGAGCGCCGAGTTC
TTAGAAGGAATCCCAACTTTGGTACCCTCGGATGAGAAGAGAAAGCTGTACATGGGCACCGGGAGGAATGACACGTTCACA
TACGCTGGATGCCAGGGGCTAACTAAGCCGAAGGTACAAATAGTGTTGGACCACAACACCCAAGTGTGTAGCGCGAATGTG
ATGTACACGGCACTTTCTAGAGCCACCGATAGGATTCACTTCGTGAACACAAGTGCAAATTCCTCTGCCTTCTGGGAAAAG
TTGGACAGCACCCCTTACCTCAAGACTTTCCTATCAGTGGTGAGAGAACAAGCACTCAGGGAGTACGAGCCGGCAGAGGCA
GAGCCAATTCAAGAGCCTGAGCCCCAGACACACATGTGTGTCGAGAATGAGGAGTCCGTGCTAGAAGAGTACAAAGAGGAA
CTCTTGGAAAAGTTTGACAGAGAGATCCACTCTGAATCCCATGGTCATTCAAACTGTGTCCAAACTGAAGACACAACCATT
CAGTTGTTTTCGCATCAACAAGCAAAAGATGAGACCCTCCTCTGGGCGACTATAGATGCGCGGCTCAAGACCAGCAATCAA
GAAACAAACTTCCGAGAATTCCTGAGCAAGAAGGACATTGGGGACGTTCTGTTTTTAAACTACCAAAAAGCTATGGGTTTA
CCCAAAGAGCGTATTCCTTTTTCCCAAGAGGTCTGGGAAGCTTGTGCCCACGAAGTACAAAGCAAGTACCTCAGCAAGTCA
AAGTGCAACTTGATCAATGGGACTGTGAGACAGAGCCCAGACTTCGATGAAAATAAGATTATGGTATTCCTCAAGTCGCAG
TGGGTCACAAAGGTGGAAAAACTAGGTCTACCCAAGATTAAGCCAGGTCAAACCATAGCAGCCTTTTACCAGCAGACTGTG
ATGCTTTTTGGAACTATGGCTAGGTACATGCGATGGTTCAGACAGGCTTTCCAGCCAAAAGAAGTCTTCATAAACTGTGAG
ACCACGCCAGATGACATGTCTGCATGGGCCTTGAACAACTGGAATTTCAGCAGACCTAGCTTGGCTAATGACTACACAGCT
TTCGACCAGTCTCAGGATGGAGCCATGTTGCAATTTGAGGTGCTCAAAGCCAAACACCACTGCATACCAGAGGAAATCATT
CAGGCATACATAGATATTAAGACTAATGCACAGATTTTCCTAGGCACGTTATCAATTATGCGCCTGACTGGTGAAGGTCCC
ACTTTTGATGCAAACACTGAGTGCAACATAGCTTACACCCATACAAAGTTTGACATCCCAGCCGGAACTGCTCAAGTTTAT
GCAGGAGACGACTCCGCACTGGACTGTGTTCCAGAAGTGAAGCATAGTTTCCACAGGCTTGAGGACAAATTACTCCTAAAG
TCAAAGCCTGTAATCACGCAGCAAAAGAAGGGCAGTTGGCCTGAGTTTTGTGGTTGGCTGATCACACCAAAAGGGGTGATG
AAAGACCCAATTAAGCTCCATGTTAGCTTAAAATTGGCTGAAGCTAAGGGTGAACTCAAGAAATGTCAAGATTCCTATGAA
ATTGATCTGAGTTATGCCTATGACCACAAGGACTCTCTGCATGACTTGTTCGATGAGAAACAGTGTCAGGCACACACACTC
ACTTGCAGAACACTAATCAAGTCAGGGAGAGGCACTGTCTCACTTTCCCGCCTCAGAAACTTTCTTTAACCGTTAAGTTAC
CTTAGAGATTTGAATAAGATGGATATTCTCATCAGTAGTTTGAAAAGTTTAGGTTATTCTAGGACTTCCAAATCTTTAGAT
TCAGGACCTTTGGTAGTACATGCAGTAGCCGGAGCCGGTAAGTCCACAGCCCTAAGGAAGTTGATCCTCAGACACCCAACA
TTCACCGTGCATACACTCGGTGTCCCTGACAAGGTGAGTATCAGAACTAGAGGCATACAGAAGCCAGGACCTATTCCTGAG
GGCAACTTCGCAATCCTCGATGAGTATACTTTGGACAACACCACAAGGAACTCTAACCAGGCACTTTTTGCTGACCCTTAT
CAGGCACCGGAGTTTAGCCTAGAGCCCCACTTCTACTTGGAAACATCATTTCGAGTTCCGAGGAAAGTGGCAGATTTGATA

GCTGGCTGTGGCTTCGATTTCGAGACCAACTCACCGGAAGAAGGGCACTTAGAGATCACTGGCATATTCAAAGGGCCCCTA
CTCGGAAAGGTGATAGCCATTGATGAGGAGTCTGAGACAACACTGTCCAGGCATGGTGTTGAGTTTGTTAAGCCCTGCCAA
GTGACGGGACTTGAGTTCAAAGTAGTCACTATTGTGTCTGCCGCACCAATAGAGGAAATTGGCCAGTCCACAGCTTTCTAC
AACGCTATCACCAGGTCAAAGGGATTGACATATGTCCGCGCAGGGCCATAGGCTGACCGCTCCGGTCAATTCTGAAAAAGT
GTACATAGTATTAGGTCTATCATTTGCTTTAGTTTCAATTACCTTTCTGCTTTCTAGAAATAGCTTACCCCACGTCGGTGA
CAACATTCACAGCTTGCCACACGGAGGAGCTTACAGAGACGGCACCAAAGCAATCTTGTACAACTCCCCAAATCTAGGGTC
ACGAGTGAGTCTACACAACGGAAAGAACGCAGCATTTGCTGCCGTTTTGCTACTGACTTTGCTGATCTATGGAAGTAAATA
CATATCTCAACGCAATCATACTTGTGCTTGTGGTAACAATCATAGCAGTCATTAGCACTTCCTTAGTGAGGACTGAACCTT
GTGTCATCAAGATTACTGGGGAATCAATCACAGTGTTGGCTTGCAAACTAGATGCAGAAACCATAAGGGCCATTGCCGATC
TCAAGCCACTCTCCGTTGAACGGTTAAGTTTCCATTGATACTCGAAAGAGGTCAGCACCAGCTAGCCCGGGATGATCAAGG
TGTACAGGTACGAGATCGTGAAGCCTCTTGACCTCGACTGGAAAGAGTTCGGAACTATCCTTAGGCAGCTCCAGCAAGAGA
CTAGGTTCGCTCTTAACAAGGCTACTCAGCTTGCTTGGGAGTGGATGGGATTCAGCTCTGACTACAAGGATAACCACGGGG
AGTACCCGAAGTCTAAGGACATCCTCGGATACACTAACGTGCACGGATACGCTTACCACACCATCAAGACCAAGGCTTACA
GGCTCAACAGCGGAAACCTCTCTCAGACTATCAAGAGGGCTACCGACAGATTCAAGGCCTACCAGAAAGAGATCCTCCGAG
GGGATATGAGCATCCCGTCTTACAAGAGAGATTATCCGCTCGACCTCATCAAAGAGAACATCAGCGTCAACCGTATGAACC
ACGGCGACTATATCGCTAGCCTCTCTTTGCTTTCTAACCCGGCCAAGCAAGAGATGAACGTGAAGCGTAAGATCAGCGTGA
TCATCATCGTGCGTGGTGCTGGAAAGACCATCATGGATCGTATCCTTAGCGGCGAGTACCAGGTTTCAGCTTCTCAGATCA
TCCACAAGGACCGTAAGAACAAGTGGTACTTGAACATCTCCTACAGATTCGAGCCTCAGACCAGAGTTCTCGATCTCAACA
AGATCATGGGGATCGATCTCGGAGTTGCTGTGGCTGCTTACATGGCTTTCCAACACACCCCTGCTAGGTACAAGCTTGAAG
GTGGTGAAATTGAGAACTTCAGGCGTCAGGTTGAGAGCCGTAGAATCTCTATGCTCAGGCAGGGAAAGTATGCTGGTGGTG
CTAGAGGTGGACACGGAAGAGATAAGAGGATCAAGCCTATCGAGCAGCTCAGGGACAAGATCGCTAACTTCAGAGATACCA
CCAACCACAGGTACAGCCGTTACATCGTGGACATGGCCATCAAAGAAGGATGCGGAACCATCCAGATGGAAGATCTCACCA
ACATCCGTGACATCGGGTCTAGATTCCTCCAGAACTGGACCTACTACGACCTCCAGCAGAAGATCATCTACAAGGCTGAAG
AGGCCGGCATCAAGGTGATCAAGATTGATCCTCAGTACACCAGCCAGAGGTGTTCTGAGTGTGGAAACATCGACTCCGGAA
ACAGAATCGGCCAGGCTATCTTCAAGTGTCGTGCTTGTGGATACGAGGCTAACGCTGATTACAACGCCGCTAGGAACATTG
CGATCCCGAACATCGACAAGATTATCGCCGAGAGCATCAAGTCAAGGGCTGATCCTAAGAAGAAGAGGAAGGTTTGAACGC
GTGGATTCGTCGGTTCAGCGACGATAAGCCGAGAAGTGCCAATAAAACTGTTAAGTGGTTTGGTAACGCTCGGTAAGGTCC
GAAAGGAGAACCACTGAACGTAGTAGCGACTCCATGGGGAATATTATGTCTATAAATATAAGAGACCCTCTTATAGTAAGC
AGAGTTGTTGGAGACGTTCTTGATCCGTTTAATAGATCAATCACTCTAAAGGTTACTTATGGCCAAGTCGACCGCCGATGA
ACGGTTAAGTTTCCATTGATACTCGAAAGATGTCAGCACCAGCTAGCACAACACAGCCCATAGGGTCAACTACCTCAACTA
CCACAAAAACTGCAGGCGCAACTCCTGCCACAGCTTCAGGCCTGTTCACCATCCCGGATGGGGATTTCTTTAGTACAGCCC
GTGCCATAGTAGCCAGCAATGCTGTCGCAACAAATGAGGACCTCAGCAAGATTGAGGCTATTTGGAAGGACATGAAGGTGC
CCACAGACACTATGGCACAGGCTGCTTGGGACTTAGTCAGACACTGTGCTGATGTAGGATCATCCGCTCAAACAGAAATGA
TAGATACAGGTCCCTATTCCAACGGCATCAGCAGAGCTAGACTGGCAGCAGCAATTAAAGAGGTGTGCACACTTAGGCAAT
TTTGCATGAAGTATGCTCCAGTGGTATGGAACTGGATGTTAACTAACAACAGTCCACCTGCTAACTGGCAAGCACAAGGTT
TCAAGCCTGAGCACAAATTCGCTGCATTCGACTTCTTCAATGGAGTCACCAACCCAGCTGCCATCATGCCCAAAGAGGGGC
TCATCCGGCCACCGTCTGAAGCTGAAATGAATGCTGCCCAAACTGCTGCCTTTGTGAAGATTACAAAGGCCAGGGCACAAT
CCAACGACTTTGCCAGCCTAGATGCAGCTGTCACTCGAGGTCGTATCACTGGAACAACAACCGCTGAGGCTGTTGTCACTC
TACCACCACCATAACTACGTCTACATAACCGACGCCTACCCCAGTTTCATAGTATTTTCTGGTTTGATTGTATGAATAATA
TAAATAAAAAAAAAAAAAAAAAAAAAAAACTAGTGGTACCGAGCTCGATCGTTCAAACATTTGGCAATAAAGTTTCTTAAG
ATTGAATCCTGTTGCCGGTCTTGCGATGATTATCATATAATTTCTGTTGAATTACGTTAAGCATGTAATAATTAACATGTA
ATGCATGACGTTATTTATGAGATGGGTTTTTATGATTAGAGTCCCGCAATTATACATTTAATACGCGATAGAAAACAAAAT
ATAGCGCGCAAACTAGGATAAATTATCGCGCGCGGTGTCATCTATGTTACTAGATCGAATTCACTGGCCGTCGTTTTGGGTA
CCGAGCTCGAATTCTTAATTAAGTAATCATGGTCATAGCTGTTTCCTGTGTGAAATTGTTATCCGCTCACAATTCCACACA
ACATACGAGCCGGAAGCATAAAGTGTAAAGCCTGGGGTGCCTAATGAGTGAGCTAACTCACATTAATTGCGTTGCGCTCAC
TGCCCGCTTTCCAGTCGGGAAACCTGTCGTGCCAGCTGCATTAATGAATCGGCCAACGCGCGGGGAGAGGCGGTTTGCGTA
TTGGAGCTTGAGCTTGGATCAGATTGTCGTTTCCCGCCTTCAGTTTAAACTATCAGTGTTTGACAGGATATATTGGCGGGT
AAAC

LB, sgRNA for AsCas12f (ΔS3-5_v7), *NbPDS* target sequence, CaMV 35S promoter, Arabidopsis codon-optimized AsCas12f (I123Y/D195K/D208R/V232A) with SV40NLS, truncated Arabidopsis *FT* sequence, NOS terminator, RB

Supplementary Table S3. Mutation frequencies in AsCas12-transformed rice calli.

Upper and lower values in each cell are mutation frequencies, and number of mutant line/total number of analyzed calli, respectively. Different letters in the "average" row indicate significant differences ($P < 0.05$, Tukey's test).

target	AsCas12f variants	Exp.1	Exp.2	Exp.3	Exp.4		Exp.5	Exp.6	Exp.7		Exp.8		average
OsTubA3	wild type	3.7%<br>(1/27)	14.3%<br>(6/42)	11.6%<br>(5/43)			6.3%<br>(4/64)						9.0%[c]<br>(*n* = 4)
	YHAM	54.5%<br>(6/11)	59.5%<br>(25/42)	64.3%<br>(27/42)	55.7%<br>(34/61)		62.5%<br>(40/64)						59.3%[b]<br>(*n* = 5)
	HAMN		74.4%<br>(29/39)	69.2%<br>(27/39)				71.9%<br>(46/64)					71.8%[ab]<br>(*n* = 3)
	YKRA	45.5%<br>(10/22)	92.9%<br>(39/42)	74.5%<br>(35/47)	84.4%<br>(54/64)		85.9%<br>(55/64)		90.6%<br>(58/64)	93.7%<br>(59/63)	93.3%<br>(56/60)	91.1%<br>(51/56)	83.5%[a]<br>(*n* = 9)
	HKRA								98.3%<br>(58/59)	86.2%<br>(50/58)	90.0%<br>(54/60)	87.0%<br>(47/54)	90.4%[a]<br>(*n* = 4)
	MKRA		93%<br>(40/43)	88.4%<br>(38/43)				87.5%<br>(56/64)					89.6%[a]<br>(*n* =3)
	enAsCas12f				29.7%<br>(19/64)	30.2%<br>(19/63)		18.8%<br>(12/64)					26.2%[c]<br>(*n* = 3)
OsDL	wild type	0%<br>(0/21)	0%<br>(0/41)	0%<br>(0/43)			0%<br>(0/64)						0%[c]<br>(*n* = 4)
	YHAM	0%<br>(0/40)	2.3%<br>(1/43)	4.4%<br>(2/45)	1.6%<br>(1/63)		1.6%<br>(1/64)						2.0%[c]<br>(*n* = 5)
	HAMN		5.6%<br>(2/36)	2.5%<br>(1/40)				4.7%<br>(3/64)					4.3%[c]<br>(*n* = 3)
	YKRA	56%<br>(14/25)	58.1%<br>(25/43)	60.5%<br>(23/38)	64.1%<br>(41/64)		59.4%<br>(38/64)		75.9%<br>(44/58)	82.0%<br>(50/61)	83.6%<br>(46/55)	84.9%<br>(45/53)	69.4%[a]<br>(*n* = 9)
	HKRA								76.7%<br>(46/60)	67.2%<br>(41/61)	64.9%<br>(37/57)	75.0%<br>(39/52)	71.0%[a]<br>(*n* = 4)
	MKRA		41.7%<br>(15/36)	37.8%<br>(14/37)				45.3%<br>(29/64)					41.6%[b]<br>(*n* = 3)
	enAsCas12f				0%<br>(0/61)	0%<br>(0/61)		0%<br>(0/64)					0%[c]<br>(*n* = 3)
